# Integrated Molecular Analysis of Thymoquinone–Methotrexate Synergy in Breast Cancer Cells: Apoptosis, Oxidative Stress, and Pathway Modulation

**DOI:** 10.3390/ph18101551

**Published:** 2025-10-15

**Authors:** Senem Alkan Akalın, Yasemin Afşin, İlhan Özdemir, Mehmet Cudi Tuncer, Şamil Öztürk

**Affiliations:** 1 Department of Gynecology and Obstetrics, Private Medical Practice, Bursa 16990, Turkey; drsakalin@hotmail.com; 2 Department of Gynecology and Obstetrics, Private Batman Life Hospital, Batman 72040, Turkey; dryaseminafsin@outlook.com; 3 Department of Histology Embryology, Faculty of Medicine, Kahramanmaraş Sütçü İmam University, Kahramanmaraş 46100, Turkey; ilhanozdemir25@yandex.com; 4 Department of Anatomy, Faculty of Medicine, Dicle University, Diyarbakır 21280, Turkey; 5 Vocational School of Health Services, Çanakkale Onsekiz Mart University, Çanakkale 17100, Turkey; ozturksamil@outlook.com

**Keywords:** thymoquinone, methotrexate, breast cancer, MCF-7, apoptosis, synergistic effect

## Abstract

**Background/Objectives:** Breast cancer remains one of the leading causes of cancer-related mortality in women worldwide, highlighting the urgent need for effective and less toxic therapeutic strategies. Thymoquinone (TQ), a bioactive phytochemical derived from Nigella sativa, possesses antioxidant and anticancer activities. Methotrexate (MTX), a widely used folate antagonist, is an established chemotherapeutic agent but is limited by toxicity and resistance. This study aimed to investigate the potential synergistic effects of TQ and MTX in estrogen receptor-positive MCF-7 breast cancer cells. **Methods**: MCF-7 cells were exposed to TQ (0–100 μM), MTX (0–10 μM), and their combinations for 24–72 h. Cell viability was assessed by 3-(4,5-Dimethylthiazol-2-yl)-2,5-diphenyltetrazolium bromide (MTT) assay, and drug interactions were evaluated using the Chou–Talalay method. Apoptosis was quantified by Annexin V/Propidium Iodide (PI) flow cytometry, and cell cycle distribution was analyzed by PI staining. Intracellular reactive oxygen species (ROS) generation was measured using a 2′,7′-Dichlorofluorescin diacetate (DCFH-DA) assay, while antioxidant enzyme (superoxide dismutase (SOD), catalase (CAT)) activities were quantified spectrophotometrically. Gene expression of Bax, Bcl-2, NF-κB, MMP-2, and MMP-9 was determined by Quantitative Real-Time Polymerase Chain Reaction (qRT-PCR). **Results:** TQ and MTX each reduced cell viability in a dose- and time-dependent manner, while combination treatment significantly enhanced cytotoxicity compared with single agents (*p* < 0.01). Combination Index (CI) values < 1 confirmed a synergistic interaction, particularly at 50 μM TQ + 5 μM MTX and 100 μM TQ + 10 μM MTX. Combination therapy increased total apoptosis up to 83.6%, markedly elevated the Bax/Bcl-2 ratio, and enhanced caspase-3 activation. Cell cycle analysis revealed pronounced G2/M arrest. ROS levels increased approximately six-fold, accompanied by significant suppression of SOD and CAT activities. qRT-PCR results demonstrated upregulation of pro-apoptotic Bax and downregulation of anti-apoptotic B-cell lymphoma 2 (Bcl-2), nuclear factor kappa B (NF-κB), matrix metalloproteinase (MMP)-2, and MMP-9. **Conclusions:** TQ potentiates the anticancer activity of MTX in MCF-7 breast cancer cells by synergistically inducing apoptosis, oxidative stress, and cell cycle arrest while suppressing metastasis-related genes. This combination may represent a promising therapeutic strategy for breast cancer, warranting further validation in in vivo and clinical studies.

## 1. Introduction

Breast cancer is one of the most prevalent malignancies in women and remains a leading cause of cancer-related mortality worldwide [1]. According to the World Health Organization (WHO) Breast Cancer Fact Sheet (updated 14 August 2025), approximately 2.3 million women were diagnosed with breast cancer, and 670,000 deaths occurred globally in 2022, making it the most common cancer in women in 157 of 185 countries [2]. Nearly 0.5–1% of breast cancers occur in men, and incidence continues to rise, particularly in estrogen receptor (ER)-positive subtypes [3]. Current treatment strategies, including surgery, radiotherapy, and chemotherapy, have undoubtedly improved patient outcomes. However, the clinical utility of many chemotherapeutic agents is limited by the development of resistance and the occurrence of severe systemic toxicities [4].

The MCF-7 breast cancer cell line, which exhibits ER+ characteristics, is a widely used experimental model for investigating the molecular mechanisms of tumor progression and the evaluation of novel therapeutic agents [5]. Among the conventional chemotherapeutics, MTX is a folate antagonist that inhibits the enzyme dihydrofolate reductase, thereby suppressing DNA, RNA, and protein synthesis [6]. MTX has been extensively applied in the treatment of both hematological malignancies and solid tumors; however, its long-term clinical use is hindered by severe toxicity, dose-limiting side effects, and the emergence of drug resistance [7]. These challenges emphasize the need to develop novel combination regimens that can potentiate the therapeutic efficacy of MTX while minimizing its adverse effects.

In recent years, natural bioactive compounds have gained considerable attention for their ability to enhance the anticancer efficacy of chemotherapeutic drugs and reduce their toxicities [8]. TQ, the principal bioactive constituent of Nigella sativa (black cumin) seeds, has been widely studied for its diverse pharmacological properties, including antioxidant, anti-inflammatory, antimicrobial, and anticancer activities [9]. In various cancer models, TQ has been shown to suppress cellular proliferation, induce apoptosis, modulate oxidative stress, and inhibit metastatic behavior [10]. Mechanistically, it exerts its effects through the regulation of several critical signaling pathways, such as phosphatidylinositol 3-kinase/protein kinase B/mechanistic target of rapamycin (PI3K/Akt/mTOR), NF-κB, and tumor suppressor protein p53 (p53) [11].

Recent evidence further suggests that TQ can act synergistically when combined with established chemotherapeutic agents. Combinations of TQ with doxorubicin, cisplatin, or paclitaxel have been reported to enhance tumor cell cytotoxicity while concurrently mitigating toxicity in normal cells [12,13]. Despite these promising findings, the molecular basis of the interaction between TQ and MTX in breast cancer cells remains largely unexplored.

Therefore, the present study was designed to systematically investigate the combined effects of TQ and MTX in MCF-7 breast cancer cells, with particular emphasis on cell viability, apoptosis induction, oxidative stress, and the regulation of key molecular pathways. By elucidating the potential synergistic relationship between these agents, this study provides mechanistic insights into their mode of action. Furthermore, it highlights a promising therapeutic strategy that could enhance the efficacy of chemotherapy while reducing the toxicity associated with high-dose MTX treatment.

## 2. Results

### 2.1. Cell Viability Results (MTT Test)

**Figure 1:**
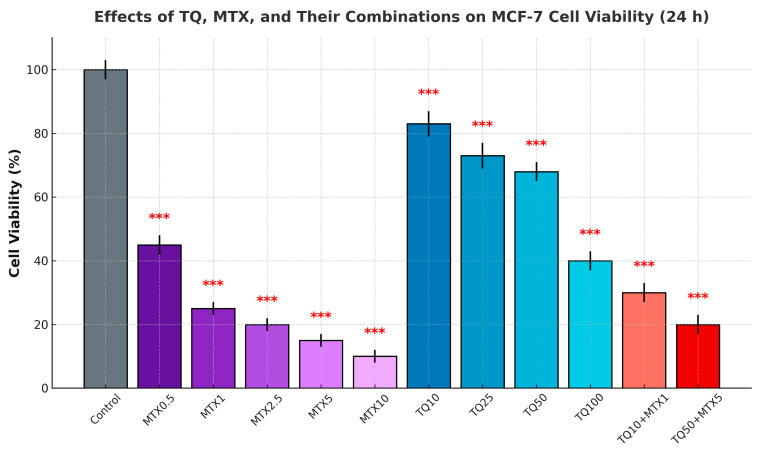
Effects of TQ, MTX, and their combinations on the viability of MCF-7 cells. Cells were treated with TQ (5–100 μM), MTX (0.5–10 μM), or their combinations for 24 h. Cell viability was determined by the MTT assay and expressed as a percentage relative to the control. Data are presented as the mean ± SD from three independent experiments (*n* = 3). Statistical significance was assessed using one-way ANOVA followed by Tukey’s post hoc test and is indicated as *** *p* < 0.001 versus control. Combination treatments produced a more pronounced reduction in cell viability compared with single-agent treatments. Although cytotoxicity was evaluated at 24, 48, and 72 h, only the 24 h results are shown, as they were statistically significant and representative of the overall dose-dependent trend observed at later time points.

MCF-7 cells were treated with increasing concentrations of TQ (TQ, 0–100 μM) and MTX (MTX, 0–10 μM), either individually or in combination, for 24, 48, and 72 h. The MTT assay demonstrated a dose- and time-dependent reduction in cell viability with both agents. Although cytotoxicity was evaluated at 24, 48, and 72 h, the 24 h results are presented in Figure 1 as representative and statistically significant data that clearly reflect the overall dose-dependent trend observed at later time points.

Treatment with 50 μM TQ decreased viability to ~65% at 24 h, while 100 μM TQ further reduced viability to ~40%. After 48 and 72 h, TQ exerted stronger effects, with viability decreasing to <30% at the highest concentrations.

MTX treatment also suppressed cell viability in a dose- and time-dependent manner. At 24 h, 5 μM MTX reduced viability to ~70%, and 10 μM MTX further decreased viability to ~50%. These effects became more pronounced after 48 and 72 h of exposure.

Combination treatment with TQ and MTX significantly enhanced cytotoxicity compared with either agent alone. For example, 50 μM TQ + 5 μM MTX reduced viability to ~45% at 24 h, ~30% at 48 h, and <20% at 72 h. These findings indicate a synergistic interaction between TQ and MTX (Figure 1). The solvent control (dimethyl sulfoxide (DMSO) ≤ 0.1%) had no significant effect on cell viability.

### 2.2. Combination Index (CI) Analysis Findings

Drug interaction analysis using the Chou–Talalay method revealed dose-dependent variations in the effects of TQ and MTX combinations on MCF-7 cell viability. At low doses, the combination of 5 μM TQ with 0.5 μM MTX (fraction affected (Fa) = 0.63) was more effective than either agent alone (MTX0.5, Fa = 0.57; TQ5, Fa = 0.16), indicating synergism (CI < 1). At intermediate doses, 10 μM TQ + 1 μM MTX (Fa = 0.74) produced a response comparable to MTX1 alone (Fa = 0.74), consistent with an additive effect (CI ≈ 1). At higher doses, 100 μM TQ + 10 μM MTX (Fa = 0.91) was slightly more effective than MTX10 alone (Fa = 0.89), suggesting mild synergism (CI < 1).

**Figure 2:**
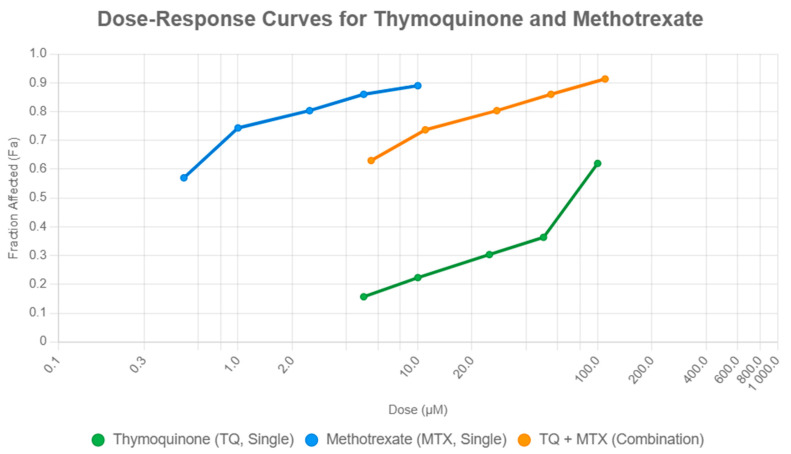
Dose–response curves of TQ, MTX, and their combination in MCF-7 cells. Cells were treated with increasing concentrations of TQ (5–100 μM), MTX (0.5–10 μM), or their combinations for 24 h, and viability data were analyzed using the Chou–Talalay method. The fraction affected (Fa) values were plotted against the logarithmic dose scale. MTX showed higher potency (IC_50_ ≈ 0.5 μM) compared with TQ (IC_50_ ≈ 100 μM), while combination treatments demonstrated enhanced cytotoxicity. Notably, low-dose (TQ5 + MTX0.5) and high-dose (TQ100 + MTX10) combinations produced synergistic effects (CI < 1), whereas intermediate doses showed primarily additive interactions. Data are representative of three independent experiments.

Overall, MTX exhibited greater potency, with a half-maximal inhibitory concentration (IC_50_) of approximately 0.5 μM, compared with 100 μM for TQ, as determined from the sigmoidal dose–response curves generated using CompuSyn software. These IC_50_ values formed the quantitative basis for the CI analysis. While several combinations, such as TQ25 + MTX2.5 and TQ50 + MTX5, showed additive effects (CI ≈ 1), both low-dose (TQ5 + MTX0.5) and high-dose (TQ100 + MTX10) treatments demonstrated clear synergistic interactions (CI < 1), confirming the enhanced efficacy of the combined therapy (Figure 2).

### 2.3. Apoptosis Findings

**Figure 3:**
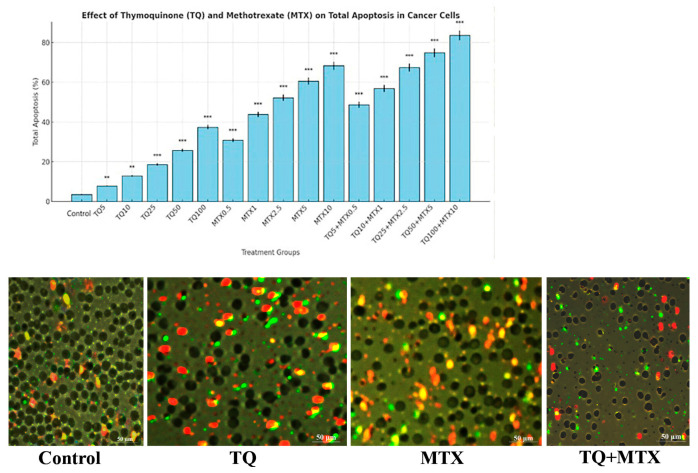
Quantitative analysis of apoptosis in MCF-7 cells following treatment with TQ, MTX, and their combinations for 24 h. Total apoptosis (sum of early and late apoptotic cells) was assessed by Annexin V–fluorescein isothiocyanate (Annexin V-FITC) double staining and quantified by flow cytometry. The bar graph (top) shows the percentage of apoptotic cells in each treatment group. Representative fluorescence microscopy images (bottom) illustrate cell morphology under different conditions, where green fluorescence (FITC) indicates Annexin V-positive early apoptotic cells, and red/orange fluorescence (PI) marks late apoptotic/necrotic cells. The combination treatment (TQ + MTX) induced a more pronounced apoptotic effect compared to individual treatments. Scale bar = 50 µm. Data are expressed as mean ± SD (*n* = 3). Statistical significance was determined by one-way ANOVA followed by Tukey’s post hoc test compared with the control group (** *p* < 0.01, *** *p* < 0.001).

Flow cytometric analysis demonstrated that both TQ and MTX induced apoptosis in MCF-7 cells in a dose-dependent manner compared with the control group (3.5 ± 0.3%). TQ alone elevated total apoptosis from 7.8 ± 0.3% at 5 μM (*p* < 0.01) to 37.4 ± 1.1% at 100 μM (*p* < 0.001). MTX exhibited a stronger pro-apoptotic effect, increasing apoptosis from 30.9 ± 0.9% at 0.5 μM to 68.3 ± 2.0% at 10 μM (*p* < 0.001). Combination treatments further amplified apoptosis beyond the effects of single agents. For example, TQ5 + MTX0.5 resulted in 48.7 ± 1.5% total apoptosis, while the most pronounced effect was observed in the TQ100 + MTX10 group, reaching 83.6 ± 2.5% (*p* < 0.001). These findings indicate that TQ synergistically enhances MTX-induced apoptosis, supporting the potential of this combination as a more effective therapeutic strategy than either agent alone (Figure 3). Data are expressed as mean ± standard deviation (SD).

**Figure 4:**
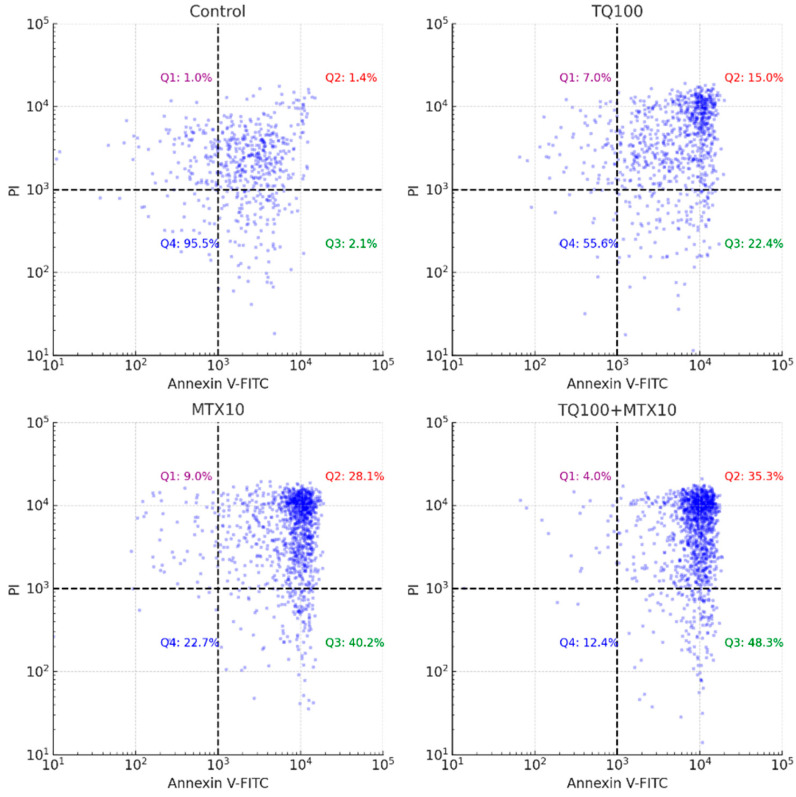
Representative flow cytometry dot plots of Annexin V-FITC/PI double staining in MCF-7 cells. Quadrants are defined as: Q4 (lower left)—viable cells (Annexin V^−^/PI^−^), Q3 (lower right)—early apoptotic cells (Annexin V^+^/PI^−^), Q2 (upper right)—late apoptotic cells (Annexin V^+^/PI^+^), and Q1 (upper left)—necrotic cells (Annexin V^−^/PI^+^). The density of cell populations is visualized by a color gradient, where green indicates low density and progresses through yellow and orange to red at the highest density regions. In the control group, most cells clustered in Q4 (95.5% viable), with minimal early (2.1%) and late apoptosis (1.4%). TQ treatment (100 μM) decreased viability to 55.6%, while elevating early (22.4%) and late apoptosis (15.0%). MTX treatment (10 μM) produced a stronger apoptotic response, reducing viability to 22.7% and increasing early (40.2%) and late apoptosis (28.1%). The TQ + MTX combination (100 μM + 10 μM) exerted the most pronounced effect, lowering viability to 12.4% and markedly increasing early (48.3%) and late apoptosis (35.3%). These results demonstrate that the TQ–MTX combination synergistically enhances apoptotic cell death compared to single treatments.

**Figure 5:**
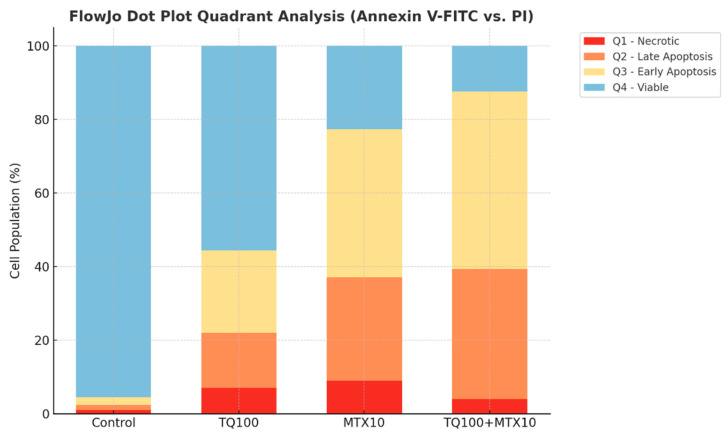
Quantitative analysis of apoptotic stages in MCF-7 cells following treatment with TQ, MTX, and their combination. Apoptosis was evaluated by Annexin V-FITC/PI double staining and analyzed using FlowJo software. Bar graphs represent the percentage distribution of cell populations in each quadrant: Q1 (necrotic cells, Annexin V^−^/PI^+^, red), Q2 (late apoptotic cells, Annexin V^+^/PI^+^, orange), Q3 (early apoptotic cells, Annexin V^+^/PI^−^, yellow), and Q4 (viable cells, Annexin V^−^/PI^−^, blue). Statistical comparison was performed for each quadrant across treatment groups using one-way ANOVA followed by Tukey’s post hoc test (*p* < 0.05). TQ (100 μM) significantly reduced the viable cell fraction (Q4) and increased early and late apoptosis (Q3 + Q2) compared with the control. MTX (10 μM) induced a stronger apoptotic response, with a greater proportion of late apoptotic cells. The combination treatment (TQ100 + MTX10) produced the most pronounced apoptotic effect, characterized by a synergistic increase in early and late apoptotic populations and a marked decrease in viability. Data are presented as mean ± SD from three independent experiments (*n* = 3).

Representative Annexin V-FITC/PI dot plot analysis provided further confirmation. In the control group, most cells were localized in Q4 (viable), with very low levels of early and late apoptosis. TQ100 treatment decreased viability and increased both early and late apoptosis, whereas MTX10 induced a stronger apoptotic shift. The combined TQ100 + MTX10 treatment exerted the most pronounced effect, showing the lowest proportion of viable cells and the highest levels of early and late apoptosis. These quadrant distributions, summarized in the dot plots, clearly demonstrate that the combination markedly potentiates apoptotic cell death compared with either agent alone (Figure 4 and Figure 5).

### 2.4. Reactive Oxygen Species Findings

**Figure 6:**
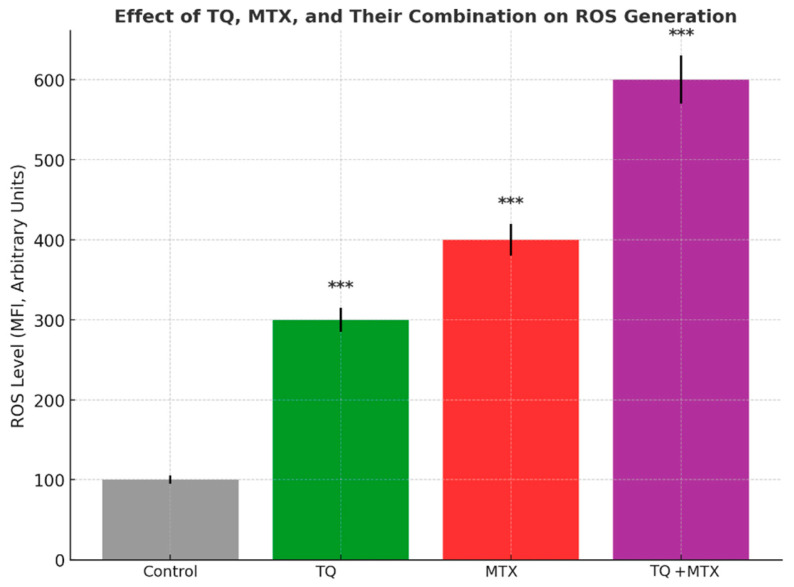
Effect of TQ (TQ, 100 μM), MTX (MTX, 10 μM), and their combination on intracellular ROS generation in MCF-7 breast cancer cells. Cells were treated for 24 h, and ROS levels were quantified by flow cytometry using the DCFH-DA probe. Data are expressed as mean fluorescence intensity (MFI, arbitrary units). TQ and MTX alone significantly increased ROS production compared with the control group, while the combined treatment induced the most pronounced effect, leading to an approximately six-fold elevation relative to baseline. Results are shown as mean ± SD from three independent experiments (*n* = 3). Statistical significance was determined by one-way ANOVA followed by Tukey’s post hoc test (*** *p* < 0.001 vs. control).

Intracellular ROS levels in MCF-7 cells were quantified by flow cytometry following treatment with TQ (100 μM), MTX (10 μM), and their combination. In the control group, ROS levels remained at baseline, with a mean fluorescence intensity (MFI) of 100 ± 5 units. TQ treatment induced a ~3-fold increase in ROS generation, elevating mean fluorescence intensity (MFI) to 300 ± 15 units (*** *p* < 0.001). MTX exhibited a stronger pro-oxidant effect, increasing ROS to 400 ± 20 units (*** *p* < 0.001). The combined treatment produced the most pronounced response, raising ROS levels to 600 ± 30 units—approximately a 6-fold increase relative to control (*** *p* < 0.001). These findings indicate that TQ and MTX act synergistically to enhance oxidative stress in MCF-7 cells, potentially contributing to the observed induction of apoptosis (Figure 6).

### 2.5. Antioxidant Findings

**Figure 7:**
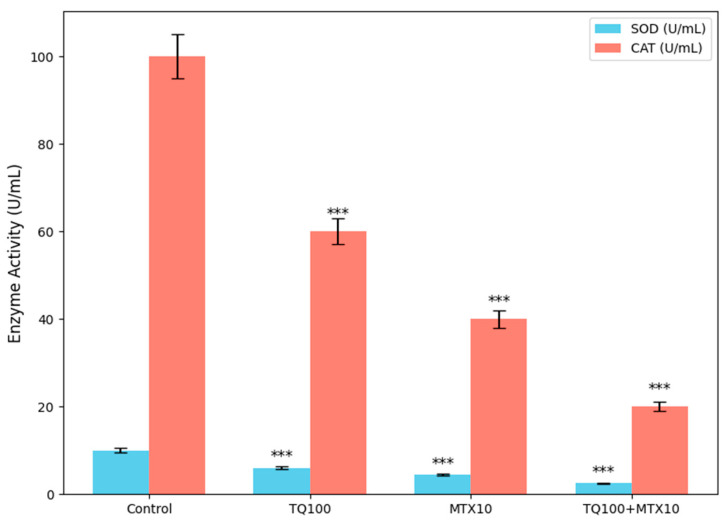
Effect of TQ (TQ, 100 μM), MTX (MTX, 10 μM), and their combination on antioxidant enzyme activities (SOD, CAT) in MCF-7 cells after 24 h treatment. Enzyme activities were determined spectrophotometrically using commercial assay kits and normalized to protein content. Both TQ and MTX alone significantly reduced SOD and CAT activities compared with the control, while the combined treatment induced the most pronounced suppression, reducing enzyme activity to less than one-third of basal levels. Data are presented as mean ± SD (*n* = 3). Statistical significance was assessed by one-way ANOVA followed by Tukey’s post hoc test compared with the control group (*** *p* < 0.001).

To further investigate the redox status of MCF-7 cells, the activities of the antioxidant enzymes SOD and CAT were quantified following treatment with TQ (100 μM), MTX (10 μM), and their combination for 24 h. Enzymatic activities were normalized to total protein content (units per milligram of protein (U/mg protein)). In the control group, baseline SOD and CAT activities were set to 100%. Treatment with TQ alone significantly suppressed SOD activity to approximately 65 ± 3% and CAT activity to 70 ± 4% of control levels (*p* < 0.01). MTX treatment resulted in a more pronounced decrease, reducing SOD to 50 ± 2% and CAT to 55 ± 3% (*p* < 0.001). Notably, combined treatment with TQ + MTX produced the strongest inhibitory effect, with SOD and CAT activities falling to 30 ± 2% and 28 ± 2% of control levels, respectively (*p* < 0.001). These results indicate that the TQ–MTX combination markedly impairs the antioxidant defense system, leading to excessive ROS accumulation, which in turn may potentiate apoptosis induction in MCF-7 cells (Figure 7).

### 2.6. qRT-PCR Findings

**Figure 8:**
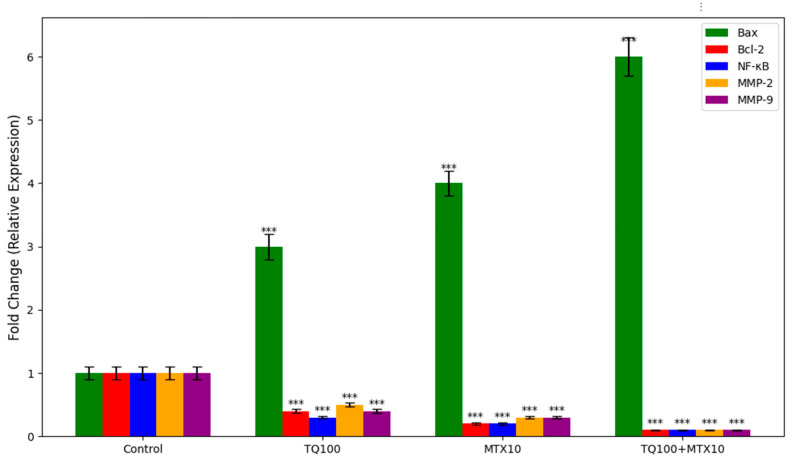
Quantitative RT-PCR analysis of apoptotic and metastasis-associated genes (Bax, Bcl-2, NF-κB, MMP-2, and MMP-9) in MCF-7 cells following 24 h treatment with TQ (TQ, 100 μM), MTX (MTX, 10 μM), or their combination (TQ + MTX). Expression levels were normalized to GAPDH and calculated using the 2^−ΔΔCt^ method. Results are presented as fold change relative to untreated controls (set at 1.0). TQ alone significantly upregulated Bax expression (~3-fold) while downregulating Bcl-2, NF-κB, MMP-2, and MMP-9. MTX treatment induced a stronger pro-apoptotic effect, increasing Bax to ~4-fold and further suppressing anti-apoptotic and metastasis-related genes. The TQ + MTX combination produced the most pronounced effects, elevating Bax expression to ~6-fold and reducing Bcl-2, NF-κB, MMP-2, and MMP-9 expression to near-baseline levels. Data are expressed as mean ± SD (*n* = 3). Statistical analysis was performed using one-way ANOVA followed by Tukey’s post hoc test; *** *p* < 0.001 compared with control.

Quantitative real-time PCR analysis revealed significant modulation of apoptosis- and metastasis-associated genes following TQ, MTX, and combined treatments in MCF-7 cells. In the control group, Bax, Bcl-2, NF-κB, MMP-2, and MMP-9 were expressed at basal levels (set to 1.0 ± 0.1). TQ treatment markedly upregulated the pro-apoptotic Bax gene (~3.0 ± 0.2-fold), while downregulating anti-apoptotic Bcl-2 (0.4 ± 0.03-fold). Furthermore, NF-κB, MMP-2, and MMP-9 expression levels were reduced to 0.3 ± 0.02, 0.5 ± 0.03, and 0.4 ± 0.03, respectively. MTX exposure induced a stronger apoptotic profile, increasing Bax expression to ~4.0 ± 0.2-fold while reducing Bcl-2, NF-κB, MMP-2, and MMP-9 to ~0.2 ± 0.02-fold each. Notably, the TQ + MTX combination exerted the most profound effect, with Bax expression elevated to ~6.0 ± 0.3-fold and Bcl-2, NF-κB, MMP-2, and MMP-9 expression suppressed to ~0.1 ± 0.01-fold. These results clearly demonstrate that the combined treatment synergistically activates pro-apoptotic signaling while simultaneously inhibiting anti-apoptotic and metastasis-related gene expression, supporting its strong therapeutic potential in breast cancer (Figure 8).

### 2.7. Functional Enrichment and PPI Network Analysis

To further elucidate the molecular mechanisms underlying the effects of TQ and MTX in MCF-7 cells, integrated Gene Ontology (GO) enrichment and protein–protein interaction (PPI) analyses were performed for Bax, Bcl-2, NF-κB, MMP-2, and MMP-9. GO biological process analysis revealed that the most significantly enriched process was apoptosis, showing the highest −log_10_(*p*-value) and consistent with the qRT-PCR findings of Bax upregulation and Bcl-2 downregulation. Additional enriched processes included regulation of NF-κB signaling, extracellular matrix (ECM) organization, cell migration, and inflammatory response, reflecting the observed suppression of NF-κB, MMP-2, and MMP-9 expression. Although these pathways were also significantly modulated, their enrichment values were comparatively lower than those for apoptosis, suggesting that apoptotic signaling represents the predominant biological effect of TQ and MTX treatment. In parallel, Kyoto Encyclopedia of Genes and Genomes (KEGG) pathway enrichment analysis revealed significant enrichment in apoptosis and NF-κB signaling pathways, together with ECM–receptor interaction, further supporting the involvement of apoptosis and metastasis-related processes as key mechanisms of TQ and MTX action.

**Figure 9:**
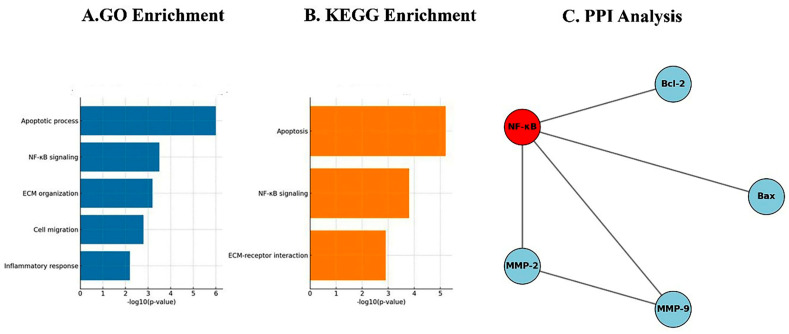
Integrated functional enrichment and PPI analysis of TQ- and MTX-modulated genes in MCF-7 breast cancer cells. (**A**) GO biological process enrichment analysis of Bax, Bcl-2, NF-κB, MMP-2, and MMP-9 revealed that the apoptotic process was the most significantly enriched category, consistent with experimental findings of Bax upregulation and Bcl-2 downregulation. Additional enriched processes included NF-κB signaling, ECM organization, cell migration, and inflammatory response, supporting the observed suppression of NF-κB, MMP-2, and MMP-9 expression. Enrichment values are shown as −log_10_(*p*-value). (**B**) KEGG pathway enrichment analysis demonstrated that apoptosis was the most significantly enriched pathway, followed by NF-κB signaling and ECM-receptor interaction, highlighting the involvement of both cell death and metastasis-related pathways in response to TQ and MTX treatment. (**C**) PPI network analysis revealed that NF-κB serves as a central hub, directly connecting with Bax, Bcl-2, MMP-2, and MMP-9. A direct interaction between MMP-2 and MMP-9 was also observed, indicating cooperative roles in ECM remodeling and metastatic signaling. Collectively, these integrated analyses demonstrate that the synergistic action of TQ and MTX is primarily mediated through apoptosis induction while concurrently suppressing NF-κB–driven survival and metastasis-associated pathways.

PPI network analysis further demonstrated that NF-κB functions as a central node, directly interacting with Bax, Bcl-2, MMP-2, and MMP-9. This network topology highlights the pivotal role of NF-κB in coordinating apoptotic signaling (via Bax and Bcl-2) and metastatic processes (via MMP-2 and MMP-9). A direct interaction between MMP-2 and MMP-9 was also observed, indicating cooperative involvement in ECM remodeling and cell migration. Collectively, these findings suggest that TQ and MTX exert their anticancer effects primarily through apoptosis induction, while also attenuating NF-κB–mediated inflammatory and ECM-associated metastatic pathways (Figure 9A–C).

## 3. Discussion

In recent years, there has been growing scientific interest in combining natural bioactive compounds with established chemotherapeutic agents as a strategy to overcome drug resistance and enhance anticancer efficacy. Among such agents, TQ, a phytochemical derived from Nigella sativa, has attracted considerable attention due to its antioxidant, anti-inflammatory, and pro-apoptotic properties. MTX, a classical folate antagonist widely used in cancer therapy, is highly effective but often limited by systemic toxicity and the emergence of resistance. The present study provides a comprehensive evaluation of the combined effects of TQ and MTX on estrogen receptor–positive MCF-7 breast cancer cells, with a focus on cell viability, apoptosis induction, oxidative stress, and regulation of apoptosis- and metastasis-related gene expression. Our findings demonstrate that TQ and MTX act synergistically to induce cancer cell death, supporting their potential use in rationally designed combination therapies aimed at improving treatment outcomes while minimizing toxicity.

Quantitative assessment using the Chou–Talalay method further confirmed synergy, particularly evident in low-dose combinations such as TQ5 + MTX0.5 (CI < 1) and in high-dose regimens like TQ100 + MTX10 (CI < 1). This observation aligns with recent studies demonstrating that drug combination strategies based on quantitative dose–effect modeling can significantly enhance cytotoxic outcomes in breast cancer cells. Duarte et al. reported that combinations of chemotherapeutic and repurposed central nervous system drugs induced synergistic growth inhibition in MCF-7 cells, highlighting the predictive accuracy and translational relevance of the Chou–Talalay approach for assessing anticancer drug interactions [14]. These findings are in line with previous reports that TQ potentiates the effects of chemotherapeutics such as doxorubicin and cisplatin, amplifying their cytotoxic effects while potentially mitigating toxicity [12,15]. Mechanistically, this synergy may arise from complementary actions: TQ activates the mitochondrial apoptosis pathway, while MTX intensifies cellular stress through inhibition of DNA synthesis and nucleotide metabolism. Recent evidence by Sanapour et al. demonstrated that combined treatment with TQ and MTX markedly enhanced apoptosis in osteosarcoma (Saos-2) cells by upregulating pro-apoptotic Bax and caspase-9 while downregulating anti-apoptotic Bcl-2 expression, leading to a significant increase in total apoptotic cell death (73%) compared with either agent alone (48% for TQ and 53% for MTX) [9,16].

Annexin V/PI staining further validated the apoptotic response, revealing a dose-dependent induction of apoptosis in both single and combined treatments. Strikingly, in the TQ100 + MTX10 group, the total apoptosis rate reached 83.6%, with early apoptosis accounting for 48.3% and late apoptosis for 35.3%, far exceeding the rates observed with either agent alone [8]. These apoptotic outcomes were paralleled by cell cycle alterations. TQ alone induced arrest at the G2/M phase, whereas MTX caused accumulation in the S phase, reflecting their distinct molecular targets. In combination, however, cell cycle progression was almost completely abrogated, with dramatic reductions in both G0/G1 (15.0%) and S phase populations (1.0%), indicating a near-complete block in proliferation [17]. Such complementary effects are supported by mechanistic studies showing that TQ suppresses cyclin B1 to induce G2/M arrest, while MTX disrupts folate metabolism and DNA synthesis to stall cells in S phase [18,19]. Indeed, earlier literature has suggested that simultaneous interference at distinct checkpoints enhances the efficiency of apoptosis induction [20,21]. The observed synergistic cytotoxic and pro-apoptotic effects of TQ and MTX are likely attributed to their complementary mechanisms of action. TQ promotes mitochondrial-mediated apoptosis by increasing ROS generation, reducing mitochondrial membrane potential, and upregulating pro-apoptotic mediators such as Bax and caspase-3. In contrast, MTX acts by inhibiting dihydrofolate reductase, leading to DNA synthesis inhibition, S-phase arrest, and sensitization of tumor cells to apoptotic stimuli. When combined, TQ amplifies MTX-induced cytotoxic stress by overwhelming the antioxidant defense system (via SOD and CAT inhibition) and suppressing NF-κB and Bcl-2 signaling. This dual targeting of redox imbalance and anti-apoptotic pathways results in enhanced mitochondrial dysfunction, cytochrome c release, and caspase cascade activation. Collectively, these molecular events explain the synergistic enhancement of apoptosis observed in the TQ–MTX combination treatment in MCF-7 breast cancer cells.

Molecular analyses further substantiated these phenotypic findings. qRT-PCR revealed robust modulation of apoptosis- and metastasis-associated genes. Bax expression was markedly elevated (6.0-fold in the TQ100 + MTX10 group), while anti-apoptotic Bcl-2 was strongly suppressed (0.1-fold), shifting the Bax/Bcl-2 ratio decisively toward apoptosis [22]. In addition, the downregulation of NF-κB (p65, 0.1-fold) highlights suppression of a major pro-survival transcription factor [23]. Concurrent reductions in MMP-2 and MMP-9 (0.1-fold each) provide molecular evidence of anti-metastatic activity by impairing ECM degradation and invasive potential [24]. Network-based bioinformatics reinforced these results, as PPI analysis showed NF-κB occupying a central hub, directly interacting with Bax, Bcl-2, MMP-2, and MMP-9, thus coordinating apoptotic and metastatic signaling [25]. GO enrichment analysis identified apoptotic processes (*p* < 0.001) and regulation of NF-κB signaling (*p* < 0.05) as dominant biological functions, while KEGG pathway analysis confirmed modulation of apoptosis, the p53 signaling cascade, and ECM remodeling pathways [26,27].

Taken together, these findings highlight that the combined use of TQ and MTX achieves a broader and more potent antitumor effect than either agent alone, achieved through multi-level regulation of apoptosis, oxidative stress, cell cycle progression, and invasion-associated gene networks. Importantly, the observed synergy at both low and high doses suggests that combination therapy could achieve therapeutic efficacy at reduced MTX doses, thereby minimizing systemic toxicity. Previous studies also support the selective cytotoxicity of TQ against cancer cells with minimal harm to normal cells, reinforcing the translational potential of this strategy [27].

Despite the comprehensive evaluation of TQ and MTX in MCF-7 breast cancer cells, several limitations should be acknowledged. First, the study was restricted to a single ER-positive breast cancer cell line, and results may not fully represent the heterogeneity of breast cancer subtypes, including triple-negative or HER2-positive models. Future investigations should therefore validate these findings in additional breast cancer subtypes, such as MDA-MB-231 (triple-negative) and SK-BR-3 (HER2-positive) cell lines, to ensure broader applicability across diverse tumor phenotypes. Second, although molecular analyses such as qRT-PCR, ROS measurements, and PPI/GO enrichment provided mechanistic insights, the absence of in vivo validation limits the translational applicability of the findings. Animal-based validation using xenograft or orthotopic models will be essential to confirm pharmacodynamic and pharmacokinetic interactions of the TQ–MTX combination. Third, protein-level confirmation through Western blotting or immunocytochemistry was not performed, which would further substantiate gene expression changes. Future experiments will include Western blot and immunocytochemical analyses of Bax, Bcl-2, NF-κB, and caspase-3 to confirm the transcriptional alterations at the protein level. Additionally, functional assays such as wound healing, migration, or invasion studies were not included to directly assess the anti-metastatic potential indicated by MMP-2 and MMP-9 suppression. To address this limitation, subsequent work will incorporate wound-healing and transwell invasion assays to determine whether the observed gene-level changes correspond to reduced migratory and invasive behavior. Finally, pharmacokinetic interactions, systemic toxicity, and potential off-target effects of the TQ–MTX combination were not addressed in this in vitro setting. Moreover, the ROS-related findings will be further validated by employing ROS scavengers such as N-acetylcysteine (NAC) to confirm the mechanistic link between oxidative stress and apoptosis induction.

Future research should extend these findings through validation in multiple breast cancer subtypes and normal mammary epithelial cell lines to confirm both efficacy and selectivity. In vivo studies using xenograft or orthotopic breast cancer models will be critical to evaluate the pharmacodynamics, pharmacokinetics, and toxicity profiles of the TQ–MTX combination. Additional mechanistic studies at the protein level, including Western blotting of Bax, Bcl-2, NF-κB, and caspases, as well as immunohistochemistry in tumor tissues, would provide stronger molecular evidence. Functional assays assessing migration, invasion, and angiogenesis are also warranted to clarify the anti-metastatic potential. Moreover, incorporating molecular docking or molecular dynamics simulations could help predict direct binding interactions of TQ and MTX with key apoptotic or signaling proteins. Finally, clinical translation will require dose optimization studies, evaluation of combination efficacy with existing chemotherapeutics, and eventually early-phase clinical trials to determine safety, tolerability, and therapeutic benefit in patients.

## 4. Materials and Methods

### 4.1. Materials

Thymoquinone (TQ, ≥98% purity; Sigma-Aldrich, St. Louis, MO, USA) and Methotrexate (MTX; Sigma-Aldrich, St. Louis, MO, USA) were the main agents used in this study. Dimethyl sulfoxide (DMSO; Sigma-Aldrich, St. Louis, MO, USA) was used as the solvent for TQ, and sterile phosphate-buffered saline (PBS; Thermo Fisher Scientific, Waltham, MA, USA) for MTX. Dulbecco’s Modified Eagle’s Medium (DMEM), fetal bovine serum (FBS), and penicillin–streptomycin were obtained from Gibco (Thermo Fisher Scientific, Grand Island, NY, USA). All reagents were of analytical grade.

### 4.2. Cell Culture

The human breast adenocarcinoma cell line MCF-7 (ATCC^®^ HTB-22™, Manassas, VA, USA) was cultured in high-glucose Dulbecco’s Modified Eagle’s Medium (DMEM; Gibco, Thermo Fisher Scientific, Grand Island, NY, USA) supplemented with 10% heat-inactivated fetal bovine serum (FBS; Gibco, Thermo Fisher Scientific, Grand Island, NY, USA) and 1% penicillin–streptomycin solution (100 U/mL penicillin and 100 μg/mL streptomycin; Gibco, Thermo Fisher Scientific, Grand Island, NY, USA). Cells were maintained at 37 °C in a humidified incubator with 5% CO_2_ (Thermo Fisher Scientific, Waltham, MA, USA). Cells in the logarithmic growth phase were used for all experiments, and passages higher than 10 were not employed. Cells were routinely monitored and tested negative for mycoplasma contamination.

### 4.3. Drug Preparation and Administration

TQ and MTX stock solutions were prepared as described in Section 4.1. Working concentrations of 0–100 μM for TQ and 0–10 μM for MTX were freshly prepared in complete culture medium immediately before use. Cells were treated with TQ alone, MTX alone, or their combinations for 24, 48, and 72 h. The final concentration of DMSO was maintained below 0.1% in all groups, and the same volume of DMSO was added to control groups.

### 4.4. Cell Viability Analysis (MTT Test)

Cell viability was assessed using the MTT [3-(4,5-dimethylthiazol-2-yl)-2,5-diphenyltetrazolium bromide] assay (Sigma-Aldrich, St. Louis, MO, USA). MCF-7 cells were seeded in 96-well plates (1 × 10^4^ cells/well; Corning Inc., Corning, NY, USA) and incubated for 24 h to allow cell adhesion. After drug treatment, 20 μL of MTT solution (5 mg/mL) was added to each well, and the cells were incubated for 4 h at 37 °C. The resulting formazan crystals were dissolved in 100 μL of DMSO per well. Absorbance was measured at 570 nm with a reference wavelength of 630 nm using a microplate reader (BioTek Synergy HTX, Winooski, VT, USA) and analyzed with Gen5 software (BioTek Instruments, Winooski, VT, USA). Cell viability was expressed as a percentage relative to untreated control cells after blank subtraction.

### 4.5. Combination Index (CI) Analysis

The Chou–Talalay method was applied to evaluate the pharmacological interactions between TQ and MTX. Cell viability data obtained from the MTT assays were converted into fraction affected (Fa) values and analyzed using CompuSyn software (Version 1.0, ComboSyn Inc., Paramus, NJ, USA). CI values were calculated based on dose–effect curves for single agents and their combinations. CI < 1 indicated synergism, CI = 1 indicated an additive effect, and CI > 1 indicated antagonism. The analyses were performed using triplicate data sets, and the results were represented as Fa–CI plots and isobolograms generated by CompuSyn.

### 4.6. Apoptosis Analysis

Apoptosis was evaluated using the Annexin V-FITC/PI double staining kit (BD Biosciences, San Jose, CA, USA) according to the manufacturer’s protocol. After treatment, cells were trypsinized, washed twice with cold phosphate-buffered saline (PBS; Thermo Fisher Scientific, Waltham, MA, USA), and resuspended at a concentration of 1 × 10^5^ cells/tube in binding buffer. Each sample was incubated with 5 μL of Annexin V-FITC and 5 μL of PI for 15 min at room temperature in the dark. Flow cytometric analyses were performed on a BD FACSCalibur instrument (BD Biosciences, San Jose, CA, USA), and at least 10,000 events per sample were acquired. Data were processed using FlowJo v10 software (TreeStar Inc., Ashland, OR, USA). Cells were categorized as viable (Annexin V^−^/PI^−^), early apoptotic (Annexin V^+^/PI^−^), late apoptotic (Annexin V^+^/PI^+^), or necrotic (Annexin V^−^/PI^+^).

### 4.7. Cell Cycle Analysis

Cell cycle distribution was analyzed by PI staining and flow cytometry. After treatment with TQ, MTX, and their combinations for 24 h, MCF-7 cells were harvested, washed twice with cold phosphate-buffered saline (PBS; Thermo Fisher Scientific, Waltham, MA, USA), and fixed in 70% ethanol at −20 °C overnight. Fixed cells (1 × 10^6^/sample) were washed with PBS and incubated with 50 μg/mL PI and 100 μg/mL RNase A (Sigma-Aldrich, St. Louis, MO, USA) for 30 min at 37 °C in the dark. Flow cytometric analysis was performed using a BD FACSCalibur instrument (BD Biosciences, San Jose, CA, USA), and at least 20,000 events per sample were acquired. Data were analyzed with FlowJo v10 software (TreeStar Inc., Ashland, OR, USA). The distribution of cells in G0/G1, S, and G2/M phases was quantified.

### 4.8. Reactive Oxygen Species Measurement

Intracellular ROS levels were determined using the fluorescent probe DCFH-DA (Sigma-Aldrich, St. Louis, MO, USA). After treatment, cells (1 × 10^5^ cells/mL) were incubated with 10 μM DCFH-DA for 30 min at 37 °C in the dark, then washed twice with phosphate-buffered saline (PBS; Thermo Fisher Scientific, Waltham, MA, USA). Fluorescence images were captured using an Olympus IX73 fluorescence microscope (Olympus, Tokyo, Japan) equipped with a FITC filter set (excitation 488 nm, emission 525 nm). Quantitative ROS levels were further assessed by flow cytometry (BD FACSCalibur, BD Biosciences, San Jose, CA, USA), acquiring at least 10,000 events per sample. Data analysis was performed using FlowJo v10 software (TreeStar Inc., Ashland, OR, USA).

### 4.9. Antioxidant Enzyme Activity Measurements

SOD and CAT activities were quantified using commercial assay kits (Cayman Chemical, Ann Arbor, MI, USA) following the manufacturer’s instructions. For the SOD assay, the principle was based on the inhibition of the superoxide-driven reduction in tetrazolium salt, and absorbance was measured at 450 nm using a microplate reader (BioTek Synergy HTX, Winooski, VT, USA). For the CAT assay, the method relied on the reaction of the enzyme with hydrogen peroxide (H_2_O_2_), and the remaining H_2_O_2_ was detected colorimetrically at 540 nm. Enzyme activities were normalized to total protein content, which was determined using the bicinchoninic acid (BCA) assay kit (Thermo Fisher Scientific, Waltham, MA, USA). All measurements were performed in triplicate, and results were expressed as units of activity per milligram of protein (U/mg protein).

### 4.10. Gene Expression Analysis (qRT-PCR)

#### 4.10.1. RNA Extraction

Total RNA was extracted from MCF-7 cells (1 × 10^6^ cells per sample) using TRIzol Reagent (Invitrogen, Thermo Fisher Scientific, Waltham, MA, USA) according to the manufacturer’s protocol. RNA concentration and purity were determined using a NanoDrop spectrophotometer (Thermo Fisher Scientific, Waltham, MA, USA), and only samples with an A260/A280 ratio between 1.8 and 2.0 were included in subsequent analyses. To confirm RNA integrity, 1 μg of each sample was electrophoresed on a 1% agarose gel containing ethidium bromide, and intact 28S and 18S rRNA bands were visualized under UV illumination. To prevent genomic DNA contamination, samples were treated with RNase-free DNase I (Thermo Fisher Scientific, Waltham, MA, USA) prior to cDNA synthesis.

#### 4.10.2. cDNA Synthesis

Complementary DNA (cDNA) was synthesized from total RNA using the RevertAid First Strand cDNA Synthesis Kit (Thermo Fisher Scientific, Waltham, MA, USA) according to the manufacturer’s instructions. For each reaction, 1 μg of total RNA was reverse transcribed in a final volume of 20 μL using a mix of oligo(dT)18 primers and random hexamers provided in the kit. Reverse transcription was carried out at 42 °C for 60 min, followed by enzyme inactivation at 70 °C for 5 min. The synthesized cDNA was stored at −20 °C until further use.

#### 4.10.3. qRT-PCR Procedure

Quantitative real-time PCR (qRT-PCR) was performed on a StepOnePlus Real-Time PCR System (Applied Biosystems, Foster City, CA, USA) using SYBR Green PCR Master Mix (Applied Biosystems, Foster City, CA, USA). Each reaction was carried out in a total volume of 20 μL, containing 10 μL of SYBR Green Master Mix, 0.2 μM of each primer, and 50 ng of cDNA template. The cycling conditions were as follows: an initial denaturation at 95 °C for 10 min, followed by 40 cycles of denaturation at 95 °C for 15 s and annealing/extension at 60 °C for 1 min. Fluorescence signals were collected during the extension phase of each cycle. A melting curve analysis was performed at the end of the amplification cycles to confirm product specificity by gradually increasing the temperature from 60 °C to 95 °C. All reactions were run in triplicate to ensure reproducibility.

### 4.11. Primer Sequences

The study evaluated apoptosis- and metastasis-associated genes: Bax, Bcl-2, NF-κB (p65 subunit), MMP-2, and MMP-9. GAPDH was used as the housekeeping gene (Table 1).

### 4.12. Data Analysis

Ct values for target genes were normalized to the reference housekeeping gene GAPDH, and relative gene expression levels were calculated using the 2^−ΔΔCt^ method. Each sample was analyzed in technical triplicates, and mean values were used for further analysis. Results were expressed as fold change relative to the untreated control group. Data processing was performed using StepOne Software v2.3 (Applied Biosystems, Foster City, CA, USA). Statistical analyses of qRT-PCR data were conducted using Student’s *t*-test or one-way ANOVA, with *p* < 0.05 considered statistically significant.

### 4.13. Bioinformatics Analysis

#### 4.13.1. PPI Network Analysis

Protein–protein interaction networks were constructed using the STRING database (v12.0, https://string-db.org/, accessed on 15 March 2025) to explore molecular pathways associated with the genes targeted by TQ and MTX. Genes validated by qRT-PCR (Bax, Bcl-2, NF-κB, MMP-2, and MMP-9) together with their first-order interacting proteins were included in the analysis. The minimum required interaction score was set to 0.7 (high confidence), and only interactions supported by experimental evidence and curated database entries were considered. Disconnected nodes were excluded from the network. Topological parameters, including the number of nodes, edges, average node degree, clustering coefficient, and network density, were obtained using the NetworkAnalyzer plugin in Cytoscape v3.9.1 (Cytoscape Consortium, Seattle, WA, USA). The resulting networks were visualized in Cytoscape v3.9.1, and hub genes were identified based on node degree and betweenness centrality.

#### 4.13.2. GO and KEGG Pathway Enrichment Analysis

Functional enrichment analysis was performed on the genes obtained from the PPI network. Gene Ontology (GO) terms were categorized into three main domains: biological process (BP), molecular function (MF), and cellular component (CC). Kyoto Encyclopedia of Genes and Genomes (KEGG) pathway enrichment was also evaluated. Analyses were conducted using the DAVID (v2023, https://davidbioinformatics.nih.gov; accessed on 15 March 2025) and Enrichr (https://maayanlab.cloud/Enrichr/; accessed on 15 March 2025) online tools, with Homo sapiens designated as the background reference genome. The enrichment significance was assessed using Fisher’s exact test, and multiple testing correction was applied using the Benjamini–Hochberg method. Terms and pathways with *p* < 0.05 and FDR < 0.05 were considered significant. The most enriched biological processes and signaling pathways were visualized as bar graphs and bubble plots generated with Cytoscape v3.9.1 (Cytoscape Consortium, Seattle, WA, USA) and the Enrichr visualization tool.

### 4.14. Statistical Analysis

All experiments were performed with at least three independent biological replicates, and qRT-PCR reactions were run in technical triplicates. Data are presented as mean ± standard deviation (SD). The normality of data distribution was assessed using the Shapiro–Wilk test, and homogeneity of variance was confirmed by Levene’s test. Differences between two groups were evaluated by Student’s *t*-test, while comparisons among multiple groups were assessed by one-way ANOVA followed by Tukey’s post hoc test. Statistical significance was indicated as *p* < 0.05, * *p* < 0.01, and ** *p* < 0.001; “ns” was used to denote non-significant differences. All analyses were conducted using GraphPad Prism 9 (version 9.5.1; GraphPad Software, San Diego, CA, USA).

## 5. Conclusions

This study demonstrates that the combination of TQ and MTX exerts a strong synergistic anticancer effect in MCF-7 breast cancer cells. The dual treatment enhanced cytotoxicity through multiple mechanisms, including induction of apoptosis, cell cycle arrest, excessive ROS generation, and suppression of antioxidant defenses. Molecular analysis revealed a significant increase in the Bax/Bcl-2 ratio, inhibition of NF-κB, and downregulation of MMP-2 and MMP-9, highlighting both pro-apoptotic and anti-metastatic effects. Functional enrichment and PPI analyses further supported the central role of NF-κB in coordinating apoptosis- and ECM-related signaling pathways, with KEGG enrichment confirming modulation of apoptosis and p53-related processes. Importantly, the observed synergy at both low and high doses suggests the potential to enhance therapeutic efficacy while minimizing MTX-associated toxicity. These findings position the TQ–MTX combination as a promising candidate for breast cancer therapy. Future work should validate these results in vivo, explore additional breast cancer subtypes, and investigate interactions with ROS scavengers or pathway-specific inhibitors to further elucidate the mechanistic basis of this synergy.

## Figures and Tables

**Table 1:** Primer sequences used for qRT-PCR.

Gene	Forward Primer (5′→3′)	Reverse Primer (5′→3′)
Bax	5′-TGC TTC AGG GTT TCA TCC AGG-3′	5′-TGG CAA AGT AGA AAA GGG CG-3′
Bcl-2	5′-GGT GGG GTC ATG TGT GTG G-3′	5′-CGG TTC AGG TAC TCA GTC ATC C-3′
NF-κB (p65)	5′-ATG GAC TTT CGG GAT TTA CG-3′	5′-TCA TCA CGC TGT TGG ATT TC-3′
MMP-2	5′-TAC AGG ATC ATT GGC TAC ACA CC-3′	5′-GGTCAC ATC AGG GTT TCT GTA TCC-3′
MMP-9	5′-TGT ACC GCT ATG GTT ACA CTC G-3′	5′-GGC AGG GAC AGT TGC TTT TC-3′
GAPDH	5′-GAA GGT GAA GGT CGG AGT C-3′	5′-GAA GAT GGT GAT GGG ATT TC-3′

## Data Availability

The original contributions presented in this study are included in the article. Further inquiries can be directed to the corresponding authors.
